# Influence of tidal volume on pulse pressure variation and stroke volume variation during experimental intra-abdominal hypertension

**DOI:** 10.1186/s12871-015-0105-x

**Published:** 2015-09-22

**Authors:** F. Díaz, B. Erranz, A. Donoso, T. Salomon, Pablo Cruces

**Affiliations:** 1 Critical Care Division, Department of Pediatrics, University of Alabama at Birmingham, Alabama, USA; 2 Unidad de Cuidados Intensivos Pediatricos, Clínica Alemana de Santiago, Santiago, Chile; 3 Centro de Medicina Regenerativa, Facultad de Medicina, Clínica Alemana-Universidad del Desarrollo, Santiago, Chile; 4 Unidad de Paciente Crítico Pediátrico, Hospital Clínico Metropolitano La Florida, Santiago, Chile; 5 Centro de Investigación de Medicina Veterinaria, Escuela de Medicina Veterinaria, Facultad de Ecología y Recursos Naturales, Universidad Andres Bello, Santiago, Chile; 6 Pediatric ICU, Hospital El Carmen de Maipú, Camino a Rinconada 1201, Maipú, Santiago, Chile

## Abstract

**Background:**

Pulse pressure variation (PPV) and stroke volume variation (SVV) are frequently used to assess fluid responsiveness in critically ill patients on mechanical ventilation (MV). There are many factors, in addition to preload that influence the magnitude of these cyclic variations. We sought to investigate the effect of tidal volume (V_T_) on PPV and SVV, and prediction of fluid responsiveness in a model of intra-abdominal hypertension (IAH).

**Methods:**

Twelve anesthetized and mechanically ventilated piglets on continuous pulse contour cardiac output monitoring. Hypovolemia was ruled out with 2 consecutive fluid boluses after instrumentation. IAH was induced by intraperitoneal instillation of colloid solution with a goal of reducing respiratory system compliance by 50 %. Subjects were classified as fluid responders if stroke volume increased >15 % after each fluid challenge. SVV and PPV were recorded with tidal volumes (V_T_) of 6, 12 and 18 ml/kg before IAH after IAH induction and after a fluid challenge during IAH.

**Results:**

V_T_ influenced PPV and SVV at baseline and during IAH, being significantly larger with higher V_T_. These differences were attenuated after fluid administration in both conditions. After IAH induction, there was a significant increase in SVV with the three-tested V_T_, but the magnitude of that change was larger with high V_T_: with 6 ml/kg from 3 % (3, 4) to 5 % (4, 6.25) (*p* = 0.05), with 12 ml/kg from 5 % (4, 6) to 11 % (8.75, 17) (*p* = 0.02) and 18 ml/kg from 5 % (4,7.5) to 15 % (8.75, 19.5) (*p* = 0.02). Similarly, PPV increased with all the tested V_T_ after IAH induction, being this increase larger with high V_T_: with 6 ml/kg from 3 % (2, 4.25) to 6 % (4.75, 7) (*p* = 0.05), with 12 ml/kg from 5 % (4, 6) to 13.5 % (10.25, 15.5) (*p* = 0.02) and 18 ml/kg from 7 % (5.5, 8.5) to 24 % (13.5, 30.25) (*p* = 0.02). One third of subjects responded to fluid administration after IAH, but neither SVV nor PPV were able to identify the fluid responders with the tested V_T_.

**Conclusion:**

IAH induction in non-hypovolemic subjects significantly increased SVV and PPV with the three tested V_T_, but the magnitude of that change was higher with larger V_T_. This observation reveals the dependence of functional hemodynamic markers on intrathoracic as well intra-abdominal pressures, in addition to volemic status. Also, PPV and SVV were unable to predict fluid responsiveness after IAH induction. Future studies should take into consideration these findings when exploring relationships between dynamic preload indicators and fluid responsiveness during IAH.

**Electronic supplementary material:**

The online version of this article (doi:10.1186/s12871-015-0105-x) contains supplementary material, which is available to authorized users.

## Background

Functional hemodynamic monitoring has been demonstrated to be a powerful tool in critically ill patients. Careful selection of patients that will respond to fluid administration may help avoid fluid overload. Functional hemodynamic monitoring including stroke volume variation (SVV) and pulse pressure variation (PPV), have been shown to be more accurate in predicting fluid responsiveness than classically used static parameters (central venous pressure (CVP) and pulmonary artery occlusion pressure) in mechanically ventilated patients without spontaneous breathing [1–3].

These dynamic indexes are based on cyclic transmission of airway pressure to the pleural and pericardial spaces, which induces changes in venous return and preload. Due to the complex relationship between intrathoracic structures (heart-lung interactions) numerous studies have demonstrated that functional hemodynamic monitoring parameters do not only depend on preload and ventricular interdependence. Mechanical ventilation (MV) settings like tidal volume (V_T_), PEEP, driving pressure and respiratory rate (RR) as well as lung compliance are potential factors that may decrease the ability of functional hemodynamic monitoring parameters to predict fluid responsiveness [4–6].

Abdominal Compartment Syndrome (ACS) represents the final stage of a pathologic process caused by an increase in intra-abdominal pressure (IAP) to a degree that can compromise regional blood flow. It has significant morbidity, related to the ongoing inflammation due to ischemia and organ dysfunction, ultimately causing death [7, 8]. Judicious fluid administration plays a key role in the management of ACS. Early fluid therapy is fundamental during the initial resuscitation phase, but its liberal use is a known risk factor for ACS and excessive fluid administration may worsen IAP [7–12]. Therefore functional hemodynamic monitoring has been proposed to guide fluid therapy in patients at risk of ACS and during increased IAP [10, 13, 14].

Cyclic changes in SV and PP induced by positive pressure ventilation can be affected by extra thoracic modifications in compliance, such as ACS or contained laparotomy [15, 16]. Current literature has yielded conflicting data regarding the ability of PPV and SVV to predict fluid responsiveness in subjects with increased IAP [17–22]. This shows the complexity of the relationship between IAP and intrathoracic pressure; as well as the effect of pressure transmitted to intrathoracic vascular structures during MV [5, 7, 8, 23]. In addition protective ventilatory strategies [24, 25], specifically small V_T_, have been shown to modify functional hemodynamic monitoring parameters when lung compliance is reduced, like ARDS, due to low transmission of pressure from airway to pleura [4–6, 26, 27]. However the effect of different V_T_ on functional hemodynamic monitoring in conditions of reduced respiratory system compliance due to extrapulmonary causes has not been studied. We sought to investigate the effect of different V_T_ on PPV and SVV in a model of intra-abdominal hypertension. We hypothesize that acute increase in IAP increases SVV and PPV readings in euvolemic animals, and this increase is proportional to the size of V_T_.

## Methods

The experimental protocol was approved by Facultad de Medicina Clínica Alemana-Universidad del Desarrollo Ethics Committee and the CONICYT (Comisión Nacional de Investigación Científica y Tecnológica) Bioethics Advisory Committee. All of the experimental procedures were consistent with the Guiding Principles in the Care and Use of Laboratory Animals adopted by the American Physiological Society.

### Animal preparation and anesthesia

12 anesthetized Large-White piglets (4.9 ± 0.05 kg) were used in this study. Animals were premedicated with intramuscular acepromazine (1.1 mg/kg) and ketamine (20 mg/kg). The trachea was cannulated with a 3.5-mm (internal diameter) cuffed tracheostomy tube (Mallinckrodt Shiley, St. Louis, MO), the left jugular vein with a 4 F double lumen catheter (Arrow, Reading, PA, USA) and the right femoral artery with a 4 F thermistor-tipped catheter (PV2014L08; Pulsion Medical Systems, Munich, Germany), all via cut down. A peritoneal dialysis catheter was inserted with Seldinger Technique under aseptic conditions. Animals were ventilated in a volume control mode (EVITA XL®, Dräger Medical, Lübeck, Germany). Initial settings were V_T_ 10 ml/kg, PEEP 5 cmH_2_O, respiratory rate (RR) 20 breaths per minute (bpm), inspiratory time 0.75 s, and FiO_2_ 0.5. Anesthesia and neuromuscular blockade were maintained by continuous infusion of propofol (10 mg/kg/h), fentanyl (4 μg/kg/h), and pancuronium (0.2 mg/kg/h) throughout the experiment. Hydration was maintained with a continuous infusion of normal saline at 5 ml/kg/h. The temperature was maintained at 37.2 ± 0.4 °C by conventional convective methods.

### Experimental protocol

**Fig. 1:**
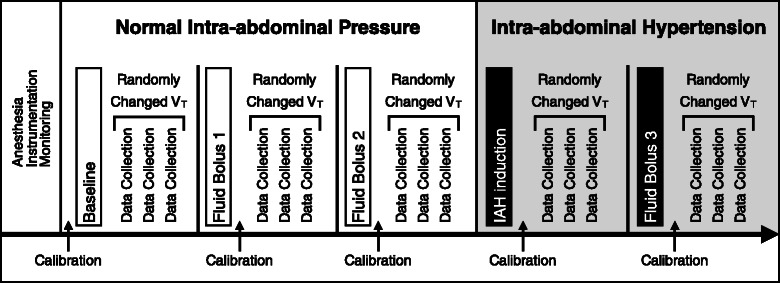
Diagram of the experimental protocol. Calibration refers to transpulmonary thermodilution for calibration of pulse contour–derived cardiac output and assessment of stroke volume variation and pulse pressure variation. Randomly changed V_T_ refers to tidal volume 6, 12 or 18 ml/kg applied in random order. (V_T_: Tidal volume, IAH, intra-abdominal hypertension)

Subjects were exposed to three different V_T_ 6, 12 and 18 ml/kg in random order during the following experimental steps: baseline, after a 20 ml/kg normal saline (NS) fluid bolus (Fluid Bolus 1), after a second 20 ml/kg NS fluid bolus (Fluid Bolus 2), after intra-abdominal hypertension (IAH) induction and after a fluid bolus of 20 ml/kg during IAH (Fluid Bolus 3) (Fig. 1). Each V_T_ was applied from 2 to 5 min allowing at least 45 s of steady reading of SVV and PPV in PiCCO® plus display (Pulsion Medical Systems, München, Germany). After testing the 3 different V_T_, subjects were placed on V_T_ 10 ml/kg until next randomization. After each fluid challenge PiCCO® plus device was re-calibrated and all measurements were obtained after 15 min of stable hemodynamic conditions.

IAH was induced by intraperitoneal instillation of Voluven® 6 % (Fresenius-Kabi, Bad Homburg, Germany) in successive aliquots of 100 ml through the peritoneal catheter. Target IAP was defined as the IAP necessary to decrease the static respiratory system compliance (C_RS_) by 50 %. We chose this target to ensure that the experimentally induced IAP was high enough to induce significant extra-abdominal organ dysfunction. At the end of the experiment the animals were euthanized.

### Measurements

Hemodynamic and respiratory measurements were performed at baseline and at the end of the experiment.

- **Pulmonary measurements:** Arterial blood gases were determined with i-STAT EG6+ Cartridges (Abbott, Princeton, NJ, USA) from blood samples drawn from the arterial catheter. Oxygenation was assessed with the PaO_2_/FiO_2_ ratio. C_RS_ was calculated as V_T_/(Ppl - PEEP_TOT_), where Ppl is the plateau pressure measured after an inspiratory hold of 4 s, and PEEP_TOT_ is the total end-expiratory airway pressure measured after an expiratory hold of 4 s. These variables were recorded from the ventilator display.
- **Hemodynamic measurements:** Heart rate (HR), mean arterial pressure (MAP), and central venous pressure were monitored with an Infinity® Delta XL monitor (Dräger Medical, Lübeck, Germany). Zero pressure was set at the midaxillary line. Cardiac index (CI) was measured with a commercially available device PiCCO® plus. According to manufacturer´s instructions, single thermal indicator transpulmonary thermodilution was performed in triplicate by injection of a 5-ml bolus of iced normal saline solution into the superior cava vein through the jugular catheter. The CI was internally computed from an analysis of the thermodilution curve with a modified Stewart-Hamilton algorithm [19]. The body surface area of the piglets was calculated as K/weight (in kg)^2/3^, where K = 0.112 for pigs.
- **Functional Hemodynamic measurements:** In the PiCCO® plus device, SVV was calculated from the mean values of four minimum and maximum stroke volumes averaged during the last 30 s (SVmean):
  $$ \mathsf{S}\mathsf{V}\mathsf{V}=\left(\mathsf{SVmax}\hbox{--} \mathsf{SV}\mathsf{m}\mathsf{i}\mathrm{n}\right)/\mathsf{S}\mathsf{V}\mathsf{m}\mathrm{e}\mathsf{a}\mathrm{n} $$
  PPV was calculated during the same time interval:
  $$ \mathsf{P}\mathsf{P}\mathsf{V}=\left(\mathsf{PPmax}\hbox{--} \mathsf{PP}\mathsf{m}\mathsf{i}\mathrm{n}\right)/\mathsf{P}\mathsf{P}\mathsf{m}\mathrm{e}\mathsf{a}\mathrm{n} $$
- **Fluid responsiveness:** At the normal intra-abdominal stage, and after IAH induction, subjects were classified as fluid responders if SVI increased greater than 15 % after the fluid bolus [5, 28].

### Statistical analysis

Data are presented as median and 25th and 75th percentile range (P25–75), unless stated. Related-Samples Wilcoxon Signed Rank test was used to assess differences between baseline data and measurements at the end of the study and Independent-Samples Mann–Whitney *U* test was used to compare data between groups. Spearman rank correlations were performed between functional hemodynamic markers and changes in SVI. Significant outliers were defined as any value more than 1.5 × interquartile range and they were excluded of the analysis.

Receiver-operating characteristic (ROC) curves were constructed to evaluate the capacity of PPV and SVV to predict fluid responsiveness under the three tested V_T_ [29].

Differences with *P* <0.05 were considered statistically significant. All statistical analyses were performed with the SPSS 20.0 software program (SPSS, Chicago, IL, USA).

## Results

All the animals completed the experimental protocol. The hemodynamic data during the experiment are presented in Table 1. Respiratory parameters with normal intra-abdominal pressure and after IAH induction are summarized in supplemental material. (Additional file 1)

**Table 1:** Hemodynamic variables at baseline, after fluid loading and after Intra-abdominal Hypertension. Presented as median and 25^th^ and 75^th^ percentile range

	Baseline	FB1	FB2	IAH	FB3
IAP	4 (3,4)			22 (19,24)^a^	
CI	4.30 (3.91,5.23)	4.66 (4.23,5.61)^a^	5.18 (4.31,5.86)^a^	4.28 (3.88,4.52)^b^	4.85 (4.04,5.16)^b^
SVI	32.0 (30.0,33.8)	39.5 (37.0,42.5)^a^	40.5 (36.0,42.5)^a^	37.5 (32.8,44.5)^a^	40.5 (39.3,47.0)^a^
HR	139 (120,180)	129 (118,155)^a^	131 (125,150)^a^	125 (118,129)^a,b^	128 (113,136)^a,b^
MAP	69 (61,72)	72 (68,82)^a^	73 (70,82)^a^	78 (75,95)^a^	90 (77,98)^a,b^
CVP	6 (6,7)	10 (8,11)^a^	11 (10,12)^a^	11 (10,12)^a^	16 (14,17)^a,b,c^
SVRI	1152 (1007,1399)	1041 (888,1276)	935 (795,1234)	1152 (1083,1408)^b^	1216 (953,1479)^b^

(*FB* fluid bolus, *IAH* intra-abdominal hypertension, *IAP* intra-abdominal pressure, mmHg, *CI* cardiac index, l/min/m^2^, *SVI* stroke volume index, mL/m^2^, *HR* heart rate, bpm, *MAP* mean arterial pressure, mmHg, *CVP* central venous pressure, mmHg, *SVRI* systemic vascular resistance index, dynes-sec/cm^−5^/m^2^)

^a^*P* <0.05 Respect to baseline

^b^*P* <0.05 Respect to fluid bolus 2 (FB2) (PRE-IAH)

^c^*P* <0.05 Respect to post-IAH

**Fig. 2:**
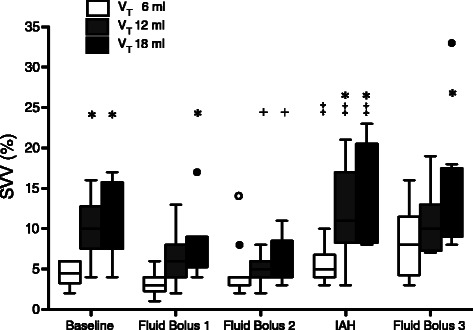
Boxplot graph of Stroke Volume Variation (SVV, %) with 6, 12 and 18 ml/kg at different steps of the experiment. Closed circles show outliers. Open circle shows outlier not included in the analysis. BL, baseline; FB1, First Fluid Bolus; FB2, Second Fluid Bolus; IAH, Intra-abdominal Hypertension; FB3, Fluid Bolus 3. *P <0.05 respect to V_T_ 6 mL/kg (in the same step). ^+^*P* <0.05 respect to BL (with equal V_T_). ^‡^*P* <0.05 respect to FB2 (normovolemic animals pre-HIA)

**Fig. 3:**
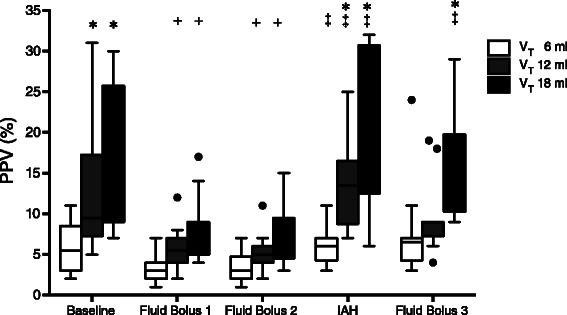
Boxplot graph of Pulse Pressure Variation (PPV, %) with 6, 12 and 18 ml/kg at different steps of the experiment. Closed circles show outliers. BL, baseline; FB1, First Fluid Bolus; FB2, Second Fluid Bolus; IAH, Intra-abdominal Hypertension; FB3, Fluid Bolus 3. *P <0.05 respect to V_T_ 6 mL/kg (in the same step). ^+^*P* <0.05 respect to BL (with equal V_T_). ^‡^*P* <0.05 respect to FB2 (normovolemic animals pre-HIA)

Before IAH induction, each successive fluid bolus increased CI, SVI, and CVP, and decreased HR (Table 1). Before fluid administration SVV and PPV were significant higher with V_T_ 12 and 18 ml/kg versus 6 ml/kg. These differences were attenuated after fluid loading (Figs. 2 and 3).

Fluid challenge resulted in 15 % or greater increase in SVI in 42 % of trials before the induction of IAH, 8 subjects after FB1 and 2 subjects after FB2 (Table 2A). Fluid responders had lower initial SVI and CVP, but there was no significant difference in CI. PPV was significantly higher in fluid responders across the range of V_T_. Of note is that this difference was more pronounced with V_T_ 18 ml/kg. SVV was significantly higher with V_T_ 12 and 18 ml/kg in fluid responders compared to non-responders, but not with V_T_ 6 ml/kg (Table 2A). There was direct correlation between SVV and the increase in SVI after fluid challenge (ΔSVI) with V_T_ 6 ml/kg (ρ = 0.4, *p* = 0.048), V_T_ 12 ml/kg (ρ = 0.57, *p* = 0.004) and 18 ml/kg (ρ = 0.64, *p* = 0.001). Also a positive correlation was found between ΔSVI and PPV with V_T_ 6 ml/kg (ρ = 0.59, *p* = 0.003), V_T_ 12 ml/kg (ρ = 0.69, *p* <0.001) and 18 ml/kg (ρ = 0.69, *p* <0.001). Scatter plot of these correlations are provided as supplemental material (Additional files 2 and Additional file 3).

**Table 2:** Hemodynamic and functional hemodynamic markers in fluid responders and non-responders before (A) and after (B) intra-abdominal hypertension induction

A.		∆SVI >15 %		*P*-value
	ALL (*n* = 24)	YES (*n* = 10)	NO (*n* = 14)	
CI (l/min/m^2^)	4.5 (4.0, 5.5)	4.5 (4.1, 5.5)	4.4 (3.9, 5.4)	0.79
∆CI (%)	5.7 (1.1, 15.1)	14.9 (3.7, 24.6)	4.7 (-8.3, 8.2)	0.106
∆SVI (%)	10.9 (2.6, 21.2)	23.2 (18.1, 33.3)	5.0 (-8.1, 9.4)	<0.001
SVI (ml/m^2^)	36.5 (32, 40)	31 (30, 39)	37 (35, 40)	0.001
SVV (%)				
V_T_ 6 ml/kg	4 (3, 6)	4 (3.8, 6)	3 (2, 5)	0.12
V_T_ 12 ml/kg	8 (6, 11)	10 (9, 12)	6.5 (4, 8)	0.011
V_T_ 18 ml/kg	9 (6, 12)	12 (9, 16)	6 (5, 9)	0.011
PPV (%)				
V_T_ 6 ml/kg	4 (3, 6)	5 (3, 7)	3 (2, 4)	0.019
V_T_ 12 ml/kg	7 (5, 11)	11 (8, 19)	6 (5, 7)	<0.001
V_T_ 18 ml/kg	9 (7, 15)	14 (9, 27)	8 (5, 9)	0.001
HR (bpm)	129 (117, 163)	139 (119, 191)	129 (114, 155)	0.045
MAP (mmHg)	69 (63, 80)	67 (63,71)	73 (64, 83)	0.214
CVP (mmHg)	7 (6, 10)	7 (6, 8)	8 (7, 11)	0.016
SVRI	1070 (895,1408)	1144 (880,1189)	1041 (913,1475)	0.837
B.		∆SVI > 15 %		*P*-value
	ALL (*n* = 12)	YES (*n* = 4)	NO (*n* = 8)	
CI (l/min/m^2^)	4.3 (3.8, 4.6)	3.8 (3.4, 4.1)	4.4 (4.2, 5.4)	0.073
∆CI (%)	8.8 (0, 19)	20.2 (13.2, 32.6)	−5.1 (-8.7, 6.4)	0.028
∆SVI (%)	6.4 (-6.5, 19.7)	26.0 (19.6, 32.0)	−4.0 (-11.4, 6.4)	0.004
SVI (ml/m^2^)	38 (33, 45)	35 (30, 40)	41 (35, 48)	0.283
SVV (%)				
V_T_ 6 ml/kg	5 (4, 7)	6 (5, 7)	5 (4, 8)	0.461
V_T_ 12 ml/kg	11 (9, 17)	17 (13, 19)	10 (7, 13)	0.154
V_T_ 18 ml/kg	15 (9, 20)	20 (18, 22)	10 (8, 16)	0.073
PPV (%)				
V_T_ 6 ml/kg	6 (5, 7)	7 (6, 9)	6 (4, 7)	0.368
V_T_ 12 ml/kg	14 (10, 16)	16 (14, 20)	12 (8, 15)	0.154
V_T_ 18 ml/kg	24 (13, 31)	28 (22, 32)	19 (11, 28)	0.214
HR (bpm)	125 (118, 131)	118 (103, 118)	128 (125, 137)	0.028
MAP (mmHg)	81 (71, 87)	80 (75, 86)	81 (68, 94)	1
CVP (mmHg)	11 (10, 13)	11 (10, 13)	11 (10, 16)	0.808
SVRI	1231 (1044,1495)	1382 (1150,1657)	1160 (985,1368)	0.368

Presented as median and 25^th^ and 75^th^ percentile range. (*CI* Cardiac index, l/min/m^2^; *SVI* stroke volume index, ml/m^2^; *SVV* stroke volume variation, %, *PPV* pulse pressure variation, %, *V*_*T*_ Tidal Volume, ml/kg, *HR* Heart rate, bpm, *MAP* mean arterial pressure, mmHg, *CVP* central venous pressure, mmHg *SVRI* systemic vascular resistance index, dynes-sec/cm^−5^/m^2^)

ROC analysis showed that PPV was a good predictor of fluid responsiveness with all V_T_. SVV had lower performance than PPV with all tested V_T_. The best cutpoint changed according the V_T_ applied, being higher with larger V_T_. (Table 3A)

**Table 3:** Receiver operating characteristics (ROC) curve statistic for predicting changes > 15 % in SVI (fluid responders) at normal intra-abdominal pressure (A) and during intra-abdominal hypertension (B)

A						
	AUC	95 % CI	*p*-value	Best cut point	Sensitivity	Specificity
V_T_ 6 ml/kg						
SVV	70 %	49–92	0.111	3.5	75 %	56 %
PPV	79 %	61–98	0.022	4.5	75 %	81 %
V_T_ 12 ml/kg						
SVV	82 %	65–99	0.012	8.5	88 %	75 %
PPV	95 %	87–100	<0.001	9.5	75 %	94 %
V_T_ 18 ml/kg						
SVV	82 %	65–99	0.013	8.5	88 %	63 %
PPV	89 %	76–100	0.002	10	88 %	81 %
B						
V_T_ 6 ml/kg						
SVV	64 %	33–95	0.445	5.5	50 %	63 %
PPV	67 %	35–99	0.35	5.5	75 %	38 %
V_T_ 12 ml/kg						
SVV	78 %	48–100	0.126	15	75 %	88 %
PPV	78 %	51–100	0.126	14.5	75 %	75 %
V_T_ 18 ml/kg						
SVV	84 %	61–100	0.062	18.5	50 %	75 %
PPV	73 %	44–100	0.203	24	75 %	63 %

(*V*_*T*_, Tidal Volume, *SVV* stroke volume variation, *PPV* Pulse pressure variation, *AUC* area under the curve)

IAP was 4 (3, 4) mmHg at baseline. The IAP of 22 (19, 23) mmHg (*p* <0.01 respect to baseline IAP) was achieved after intraperitoneal infusion of 2.5 (2.1, 2.7) l of colloid solution over 35 (30, 40) min. As planned in the experimental design, the rise of IAP caused a decrease in C_RS_ from 1.27 (1.06, 1.41) to 0.55 (0.49, 0.75) ml/cmH_2_O/kg (*p* <0.01) and PaO_2_/FiO_2_ ratio from 412 (387, 424) to 330 (290, 351) (*p* <0.01). After IAH induction there was a significant decrease in CI with no changes in CVP and HR (Table 1). Interestingly in 5 animals SVI increased (range 6 to 25 %). There was a significant increase in SVV with the three-tested V_T_, but the magnitude of that change was larger with high V_T_: with 6 ml/kg from 3 % (3, 4) to 5 % (4, 6.25) (*p* = 0.049), with 12 ml/kg from 5 % (4, 6) to 11 % (8.75, 17) (*p* = 0.02) and 18 ml/kg from 5 % (4, 7.5) to 15 % (8.75, 19.5) (*p* = 0.02) (Fig. 2). Similarly, PPV increased with all the tested V_T_, being this increase larger with high V_T_: with 6 ml/kg from 3 % (2, 4.25) to 6 % (4.75, 7) (*p* = 0.047), with 12 ml/kg from 5 % (4, 6) to 13.5 % (10.25, 15.5) (*p* = 0.02) and 18 ml/kg from 7 % (5.5, 8.5) to 24 % (13.5, 30.25) (*p* = 0.02) (Fig. 3).

Once IAH was established, SVV and PPV were significantly higher with V_T_ 12 and 18 ml/kg compared with 6 ml/kg. The differences between 6 and 12 ml/kg disappeared after the fluid bolus, but between 6 and 18 ml/kg were still significant (Figs. 2 and 3). One third of fluid bolus trials (FB3) resulted in an increase of SVI greater than 15 % during IAH. Fluid responders had lower CI and SVI, higher SVV and PPV, but these differences were not significant (Table 2B). There was not a significant correlation between ΔSVI and SVV once IAH was established with any of the tested V_T_: 6 ml/kg ρ = 0.23, *p* = 0.46; 12 ml/kg ρ = 0.46, *p* = 0.13; and 18 ml/kg (ρ = 0.56, *p* = 0.064) (Additional file 2: Figure S1B). As shown in Additional file 2: Figure S1B, there was not a significant correlation between ΔSVI and PPV after IAH induction with any of the applied V_T_: 6 ml/kg (ρ = 0.29, *p* = 0.36), V_T_ 12 ml/kg (ρ = 0.45, *p* = 0.13) and 18 ml/kg (ρ = 0.38, *p* = 0.22) (Additional file 2: Figure S1B). Scatter plot of these correlations are provided in supplemental material, Additional file 2: Figure S1. AUC showed that none of the studied variables were good predictor of fluid responsiveness during IAH (Table 3B), even adjusting for higher cutoff points.

## Discussion

The main findings of this experimental study are as follows:

i. IAH increased PPV and SVV compared to normal IAP with the three-tested V_T_, but the magnitude of these changes were larger with higher V_T_.
ii. PPV and SVV were influenced by V_T_ during IAH, being significant larger with high V_T_. These differences were attenuated after fluid administration.
iii. During IAH, neither SVV nor PPV predicted response to fluids with the three tested V_T_.

We observed significant differences in PPV and SVV with the studied V_T_ at baseline. These differences reflect the transmission of swings in the airway pressure to the vascular compartment, with greater transmission occurring at higher V_T_. After fluid challenge, CI, SVI, and static markers of intravascular fluid status increased. PPV and SVV significantly decreased with respect to baseline only under V_T_ 12 and 18 ml/kg. This observation is in accordance with previous studies that suggested that in conditions of hypovolemia fluctuations in pulse pressure and stroke volume are related to transmission of airway pressure to the vascular compartment and not the V_T_ itself [27, 30, 31]. Our findings suggest that the effect of the airway pressurization on the cyclic changes was less significant in conditions of euvolemia or hypervolemia, after the fluid administration. Low V_T_ produced variations in PP and SV, but they were very small. As shown in Fig. 1 there was a significant variation on SVV and PPV at the beginning of the experiment when high V_T_ was applied. The attenuation of this dispersion after the first and second fluid bolus also supports the change in hemodynamic condition of the subjects to a more flat portion of the Frank-Starling curve, explaining the trend towards homogeneity. We think that our experimental model may explain this, because we did not actively induce hypovolemia, animals fasted the night before the experiment, but had access to water *ad libitum*.

Forty two percent of subjects were fluid responders before IAH. Both PPV and SVV were higher in fluid responders compared to non-responders, but these differences were smaller with lower V_T_. It is important to note that when V_T_ 6 ml/kg was applied, all but one subject had PPV and SVV readings under the usual threshold to define fluid responders, leading to non-useful clinical differences, like SVV 4 (3.8, 5.3) v/s 3 (2, 5) and PPV 5 (3, 7) v/s 3 (2, 4) for responders and non-responders respectively. In accordance to previous observations, airway pressurization was not enough to demonstrate preload dependency with low V_T_ in mechanically ventilated healthy piglets [4, 6, 32, 33]. On the other hand, ROC analysis showed that PPV and SVV were good predictors of fluid response with V_T_ 12 and 18 ml/kg. PPV had better performance predicting fluid responsiveness with all the tested V_T_, being better with 12 ml/kg with a cut off of 9.5, strikingly similar to previous studies [12, 34]. Also IAH is a common problem in the intensive care unit and is responsible for significant morbidity in critically ill patients [7, 8]. Usual definitions for IAH is a sustained or repeated pathologic elevation of IAP ≥12 mmHg and ACS a sustained IAP >20 mmHg that is associated with new organ dysfunction or failure in the intensive care unit setting [7]. An important methodological difference of our experimental protocol versus previous studies was the definition of IAH. Our target was not a fixed IAP, but a significant effect on lung compliance. Surprisingly this target was achieved in accordance with current definitions of ACS, with IAP slightly greater than 20 mmHg. It has been previously reported that there is a high risk of irreversible tissue damage in with IAP >20 mmHg [7, 8].

Relationships between thoracic and abdominal compartments are complex and both have significant independent as well as interdependent influences on cardiovascular physiology. As expected, we observed a reduction in CI after induction of IAH, but without significant changes in blood pressure [33, 35]. The variable and unpredictable individual response of SVI to IAH is a practical example of the complex relationship between intra-abdominal and intrathoracic compartments. It has been described in experimental studies that moderate or small increases in IAP can increase intrathoracic blood volume, left ventricular end-diastolic area, and transmural left ventricular end-diastolic pressure, suggesting an auto-transfusion effect in euvolemic subjects, but not during hypovolemia [20, 33, 36, 37]. In our model some subjects might have been hypervolemic, that can explain that even facing IAP >20 mmHg SVI did not significantly decrease.

We found that after IAH induction, PPV and SVV increased with the 3 tested V_T_. Previous studies showed that cyclic changes in PP and SV during IAH are dependent on the pressure of abdominal compartment [5, 20, 21, 28]. Interestingly with low V_T_ (6 ml/kg) changes in SVV and PPV after IAH induction were very small and most animals were below the usual threshold described to predict fluid responsiveness. On the other hand, high V_T_ produced a large increase in SVV and PPV, showing that during IAH in addition to the pressure of the abdominal compartment, PP and SV are dependent on the airway pressure swings, and not the applied V_T_. This supports the clinical observation of Muller et al. who found that lack of significant variation of airway pressure, expressed as PIP-Ppl, in patient on mechanical ventilation with low V_T_ might limit the use of PPV to predict fluid responsiveness [27].

Fluid administration during IAH increased SVI greater than 15 % in 1/3 of the subjects. There was a non-significant trend towards higher PPV and SVV in these fluid responsive patients. ROC analysis showed that PPV and SVV were not able to discriminate between fluid responders and non-responders during IAH with the tested V_T_. It is important to note that AUC might look similar between pre-IAH and after IAH induction, but the wide confidence interval (crossing 50 %) as well as the large p-value observed, reflect that SVV and PPV did not discriminate between animals that increased their SVI greater than 15 % after the third fluid challenge. Other studies have found that SVV and PPV were useful to predict fluid responsiveness, but with different thresholds [14, 17, 19]. We think that the discrepancy of our findings is related to the experimental design. We were trying to demonstrate the complex relationship between intrathoracic and IAP and this experimental model was not designed to study hypovolemic conditions. Subjects at this stage of the experiment were euvolemic or even hypervolemic after the initial fluid challenges; surprisingly we observed a significant number of fluid responders, but SVV and PPV failed to identify them. Furthermore, in contrast to previous studies, the significant variations in SV and PP with V_T_ 12 and 18 ml/kg even in the absence of changes in SVI during fluid challenge can be explained by the large variation in intrathoracic pressures due to decreased C_RS_ in accordance with our definition of IAH.

The present study should not be understood as challenging the ability of PPV or SVV to predict fluid responsiveness. With our initial hypothesis and the obtained results we intend to point out the relationship between abdominal and thoracic compartment, considering the V_T_, airway pressurization and IAP when interpreting functional hemodynamic monitoring parameters to decide fluid administration.

Our study has some limitations. First, in addition to the interspecies physiologic differences (i.e. chest wall compliance, thorax shape and relation of intrathoracic structures [38]), we studied piglets with normal respiratory and cardiovascular function. We must be cautious extrapolating these results to critically ill patients where multiorgan involvement is frequently observed. Second, a limitation of our model is that IAH was induced over a short period of time. As most of experimental models, this one mimics acute IAH rather than the usual subacute IAH observed in the clinical setting. Our target IAP was different from previous studies, being compatible with severe ACS during critical illness and not with usual increase of IAP of pneumoperitoneum during laparoscopic surgery. Also IAH was induced after fluid challenge, limiting generalization of these observations in under-resuscitated, hypovolemic, or actively bleeding patients. Third we did not include chest wall or abdominal wall compliance measurements in our experiment, preventing the analysis of the isolated components that might have influenced SVV and PPV. Fourth, we used PiCCO® plus system to measure PPV and SVV. Although we used frequent calibration, it is not the gold standard for these measurements. Finally, the small number of subjects as well the scattered response of fluids after IAP does not allow robust receiver operating characteristic curve analysis.

## Conclusion

We found significant differences in PPV and SVV with different V_T_ at baseline and after IAH induction. These differences were attenuated after fluid administration. IAH induction in non-hypovolemic subjects significantly increased SVV and PPV with the three tested V_T_, but the magnitude of that change was higher with larger V_T_. This observation reveals the dependence of functional hemodynamic markers on intrathoracic as well intra-abdominal pressures, in addition to volemic status. In agreement with this physiological description, we found that PPV and SVV were good predictors of fluid responsiveness at baseline, but not after IAH induction, showing the complex relationship of the thoracic and abdominal compartments. Future studies should take into consideration these findings when exploring relationships between functional hemodynamic monitoring and fluid responsiveness in IAH.

Also our observations need to be studied in other hemodynamic conditions, like hypovolemia, low cardiac output and sepsis, and should include measurements of pleural pressure, chest and abdominal wall compliance, for better understanding of determinants of the dynamic markers of fluid responsiveness.

## Additional files

Additional file 1: **Respiratory parameters after instrumentation (normal intra-abdominal pressure) and after induction of intra-abdominal hypertension (IAH).** Presented as median and 25^th^ and 75^th^ percentile range. (IAP: intra-abdominal pressure; V_T_: Tidal volume, ml/kg; PIP, peak inspiratory pressure, cmH_2_O; PEEP, positive end expiratory pressure, cmH_2_O; P_PL_: Plateau pressure, cmH_2_O; C_RS_: Static Respiratory System Compliance, ml/cmH_2_O/kg.) **p* value < 0.05 respect to baseline. (DOCX 51 kb) Additional file 2: **Scatterplot showing percentage changes in stroke volume index (ΔSVI, %) and functional hemodynamic markers, Stroke Volume Variation (SVV, %) Pulse Pressure Variation (PPV, %), with the three tested tidal volumes (V**_**T**_**), 6, 12 and 18 ml/kg with normal intra-abdominal pressure.** Solid line shows regression line between variables. (PDF 53 kb) Additional file 3: **Scatterplot showing percentage changes in stroke volume index (ΔSVI, %) and functional hemodynamic markers, Stroke Volume Variation (SVV, %) Pulse Pressure Variation (PPV, %), with the three tested tidal volumes (V**_**T**_**), 6, 12 and 18 ml/kg during intra-abdominal hypertension.** Solid line shows regression line between variables. (PDF 56 kb)

## Abbreviations

ACS: Abdominal compartment syndrome
CI: Cardiac index
C_RS_: Static respiratory system compliance
CVP: Central venous pressure
IAP: Intra-abdominal pressure
IAH: Intra-abdominal hypertension
MV: Mechanical ventilation
NS: Normal saline
PEEP_TOT_: Total end-expiratory airway pressure
PPV: Pulse pressure variation
ROC: Receiver-operating characteristic
SVV: Stroke volume variation
V_T_: Tidal volume
