# Supplementary material for: Influence of tidal volume on pulse pressure variation and stroke volume variation during experimental intra-abdominal hypertension
Source: BMC Anesthesiol. 2015 Sep 22;15:127. doi: 10.1186/s12871-015-0105-x (PMC4579832; doi:10.1186/s12871-015-0105-x)
Supplement: Additional file 1: — Respiratory parameters after instrumentation (normal intra-abdominal pressure) and after induction of intra-abdominal hypertension (IAH). Presented as median and 25th and 75th percentile range. (IAP: intra-abdominal pressure; VT: Tidal volume, ml/kg; PIP, peak inspiratory pressure, cmH2O; PEEP, positive end expiratory pressure, cmH2O; PPL: Plateau pressure, cmH2O; CRS: Static Respiratory System Compliance, ml/cmH2O/kg.) *p value < 0.05 respect to baseline. (DOCX 51 kb) [file 12871_2015_105_MOESM1_ESM.docx]

**Table S1.** Respiratory parameters after instrumentation (normal intra-abdominal pressure) and after induction of intra-abdominal hypertension (IAH). Presented as median and 25^th^ and 75^th^ percentile range. (IAP: intra-abdominal pressure; V_T_: Tidal volume, ml/kg; PIP, peak inspiratory pressure, cmH_2_O; PEEP, positive end expiratory pressure, cmH_2_O; P_PL_: Plateau pressure, cmH_2_O; C_RS_: Static Respiratory System Compliance, ml/cmH_2_O/kg.) * p value < 0.05 respect to baseline.

|  | **Normal IAP** | **IAH** |
| --- | --- | --- |
| **V_T_** | 10 | 10 |
| **PIP** | 16 (16, 17.5) | 28 (23, 31)* |
| **PEEP** | 5 (5, 5) | 5 (5, 5) |
| **P_PL_** | 15 (14, 17) | 28 (22, 30)* |
| **C_RS_** | 1.27 (1.06, 1.41) | 0.55 (0.49, 0.75)* |
