# Supplementary figures and images for: Influence of tidal volume on pulse pressure variation and stroke volume variation during experimental intra-abdominal hypertension
Source: BMC Anesthesiol. 2015 Sep 22;15:127. doi: 10.1186/s12871-015-0105-x (PMC4579832; doi:10.1186/s12871-015-0105-x)

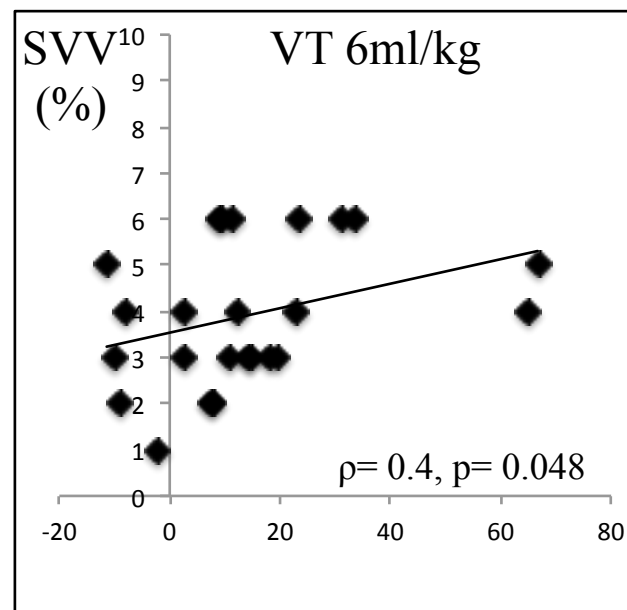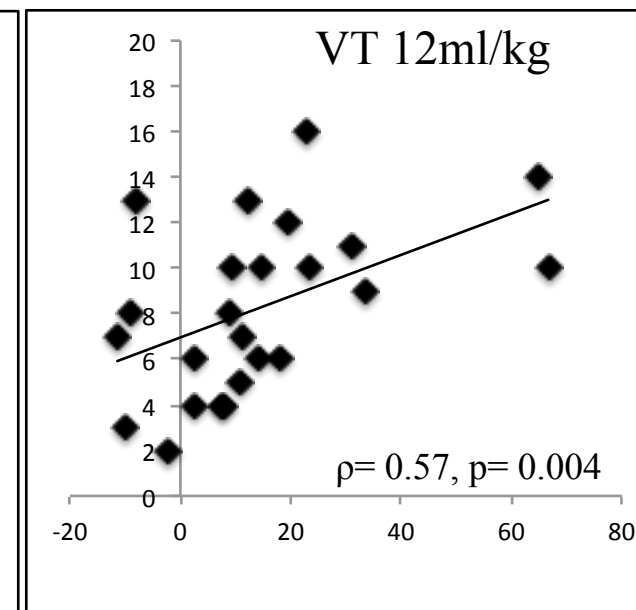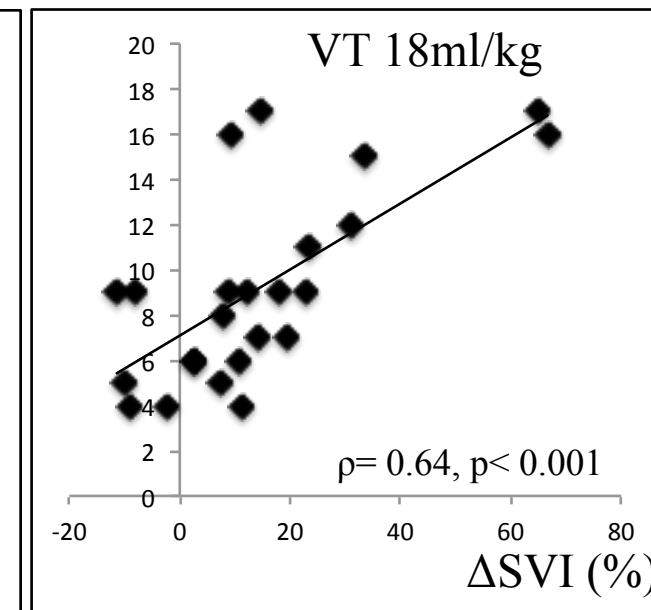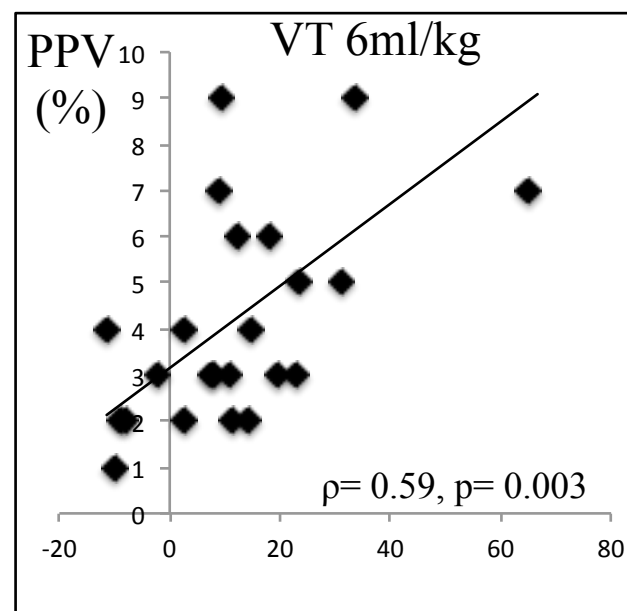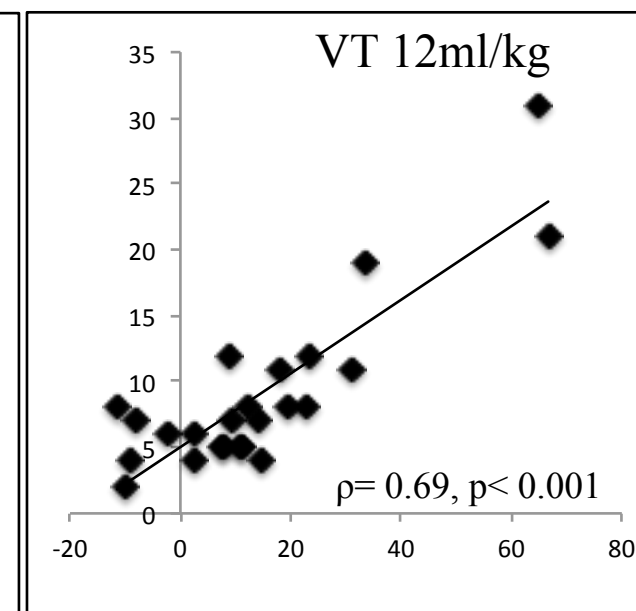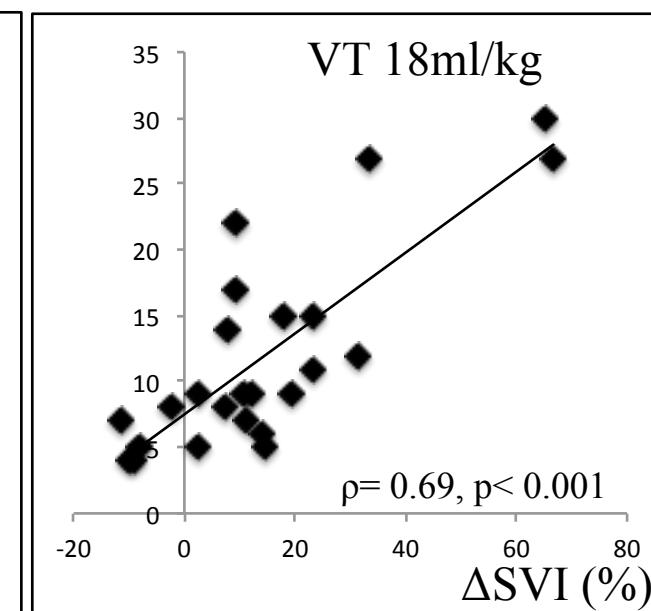

Supplement: Additional file 2: — Scatterplot showing percentage changes in stroke volume index (ΔSVI, %) and functional hemodynamic markers, Stroke Volume Variation (SVV, %) Pulse Pressure Variation (PPV, %), with the three tested tidal volumes (VT), 6, 12 and 18 ml/kg with normal intra-abdominal pressure. Solid line shows regression line between variables. (PDF 53 kb) [file 12871_2015_105_MOESM2_ESM.pdf]

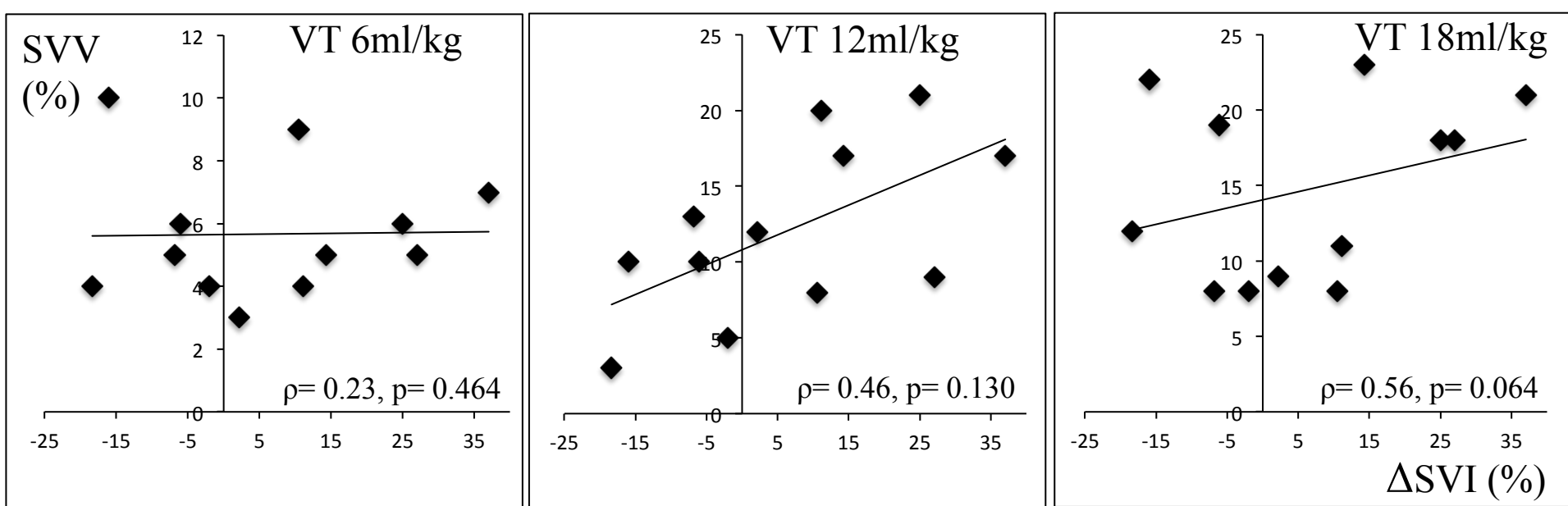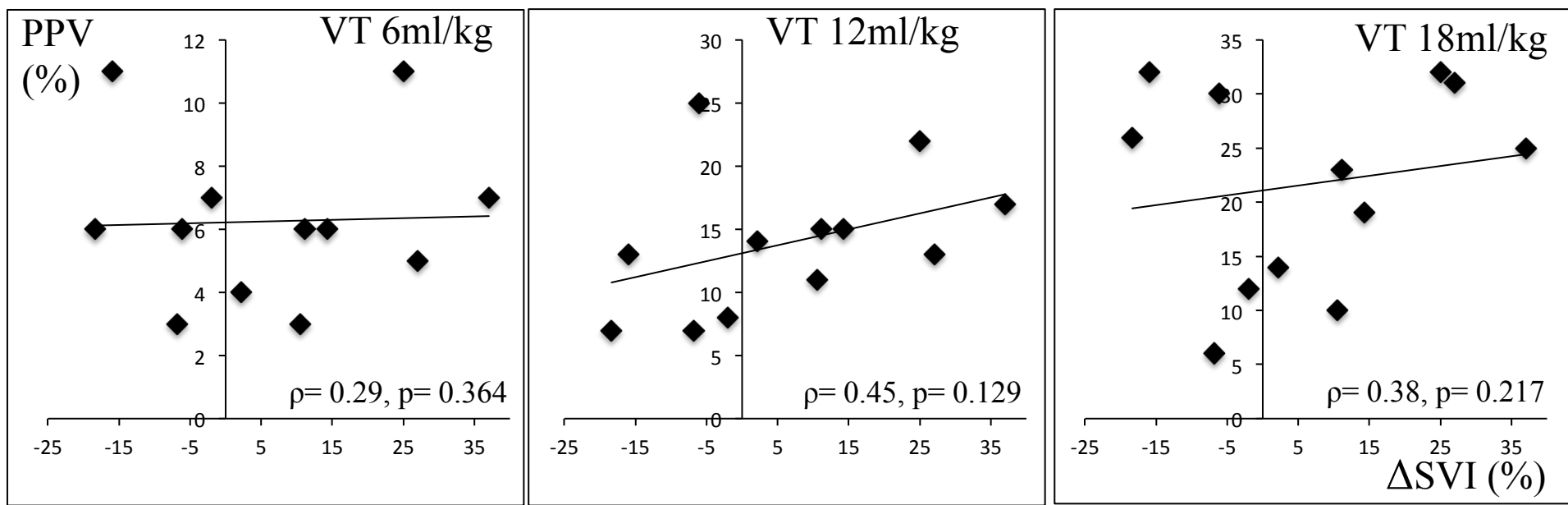

Supplement: Additional file 3: — Scatterplot showing percentage changes in stroke volume index (ΔSVI, %) and functional hemodynamic markers, Stroke Volume Variation (SVV, %) Pulse Pressure Variation (PPV, %), with the three tested tidal volumes (VT), 6, 12 and 18 ml/kg during intra-abdominal hypertension. Solid line shows regression line between variables. (PDF 56 kb) [file 12871_2015_105_MOESM3_ESM.pdf]
